# Conserved motifs in the hypervariable domain of chikungunya virus nsP3 required for transmission by *Aedes aegypti* mosquitoes

**DOI:** 10.1371/journal.pntd.0006958

**Published:** 2018-11-09

**Authors:** Giel P. Göertz, Marit Lingemann, Corinne Geertsema, Marleen H. C. Abma-Henkens, Chantal B. F. Vogels, Constantianus J. M. Koenraadt, Monique M. van Oers, Gorben P. Pijlman

**Affiliations:** 1 Laboratory of Virology, Wageningen University & Research, PB, Wageningen, The Netherlands; 2 Laboratory of Entomology, Wageningen University & Research, PB, Wageningen, The Netherlands; 3 Department of Epidemiology of Microbial Diseases, Yale School of Public Health, New Haven, CT, United States of America; University of Wisconsin Madison, UNITED STATES

## Abstract

**Background:**

Chikungunya virus (CHIKV) is a re-emerging arthropod-borne (arbo)virus that causes chikungunya fever in humans and is predominantly transmitted by *Aedes aegypti* mosquitoes. The CHIKV replication machinery consists of four non-structural proteins (nsP1-4) that additionally require the presence of a number of host proteins for replication of the viral RNA. NsP3 is essential for CHIKV replication and has a conserved macro, central and C-terminal hypervariable domain (HVD). The HVD is intrinsically disordered and interacts with various host proteins via conserved short peptide motifs: A proline-rich (P-rich) motif that has affinity for SH3-domain containing proteins and duplicate FGDF motifs with affinity for G3BP and its mosquito homologue Rasputin. The importance of these motifs for infection of mammalian cells has previously been implicated. However, their role during CHIKV infection of mosquito cells and transmission by mosquitoes remains unclear.

**Methodology / Principal findings:**

Here, we show that in-frame deletion of the P-rich motif is lethal for CHIKV replication in both mosquito and mammalian cells. However, while mutagenesis of the P-rich motif negatively affects replication both in mammalian and mosquito cells, it did not compromise the infection and transmission of CHIKV by *Ae*. *aegypti* mosquitoes. Mutagenesis of both FGDF motifs together completely inactivated CHIKV replication in both mammalian and mosquito cells. Importantly, mutation of a single FGDF motif attenuated CHIKV replication in mammalian cells, while replication in mosquito cells was similar to wild type. Surprisingly, CHIKV mutants containing only a single FGDF motif were efficiently transmitted by *Ae*. *aegypti*.

**Conclusions / Significance:**

The P-rich motif in CHIKV nsP3 is dispensable for transmission by mosquitoes. A single FGDF motif is sufficient for infection and dissemination in mosquitoes, but duplicate FGDF motifs are required for the efficient infection from the mosquito saliva to a vertebrate host. These results contribute to understanding the dynamics of the alphavirus transmission cycle and may help the development of arboviral intervention strategies.

## Author summary

Chikungunya virus (CHIKV) is a re-emerging arthropod-borne virus that is transmitted predominantly by *Aedes aegypti* mosquitoes. In 2016 alone CHIKV caused over 100.000 infections in South-America, exemplifying the impact of CHIKV disease. Previous research has suggested that the CHIKV non-structural protein 3 (nsP3) may determine the infection of mosquitoes. NsP3 is known to interact with several host proteins through a conserved proline (P)-rich and duplicate FGDF motifs that are present in its C-terminal domain. Here we investigated the importance of these conserved motifs for the infection and replication of CHIKV in both *Aedes* mosquito cells and mammalian cells. Furthermore, we assessed the role of these motifs for the transmission by *Ae*. *aegypti* mosquitoes via infectious bloodmeal experiments. We show that mutation of the P-rich motif negatively affects the replication of CHIKV in both mammalian and mosquito cells. In contrast, mutating the P-rich motif did not affect the transmission by *Ae*. *aegypti*. Mutation of both FGDF motifs together completely inactivated CHIKV in mammalian and mosquito cells, while mutation of a single FGDF motif negatively affected replication only in mammalian cells. Importantly, CHIKV containing only a single FGDF motif was still efficiently transmitted by *Ae*. *aegypti* mosquitoes. These results contribute to understanding the key interactions between alphaviruses and their mosquito vector.

## Introduction

Alphaviruses (family *Togaviridae*), such as chikungunya virus (CHIKV) and Mayaro virus, are (re-)emerging arthropod-borne (arbo)viruses that impose a global threat on human health [1]. Mayaro virus recently emerged in South America, where a large outbreak was reported in Brazil [1,2]. CHIKV is responsible for several large outbreaks in the Americas [3] and over 150.000 cases of infection were reported in the Americas in 2016 alone [4]. CHIKV disease is characterized by acute fever combined with severe arthralgia, myalgia or rash, and symptoms can last from weeks to several months [3]. Transmission of CHIKV occurs predominantly by *Aedes aegypti* mosquitoes, although some CHIKV strains have incorporated an amino acid substitution in the envelope protein (A226V) that allows transmission by *Ae*. *albopictus* [5].

Alphaviruses have a single-stranded positive-sense RNA genome that contains two open reading frames, which encode the non-structural proteins (nsP)1-4 and the structural proteins, respectively. NsP4 is the viral RNA-dependent RNA polymerase that together with nsP1-3 forms the viral replication complex. While the roles of nsP1 and nsP2 in viral replication have been extensively documented and reviewed [6–8], the exact functions of nsP3 are less clear [9,10]. During infection, nsP3 localizes partially to viral replication complexes [11,12], in accordance with the necessity of nsP3 for virus replication, as well as cytoplasmic foci [13–16]. Additionally, nsP3 interacts with several host-proteins in mammalian and mosquito cells [14,17]. Unravelling the significance and mechanisms of the interactions between nsP3 and these host-proteins in mammalian cells has been the focus of several recent studies [17–23], while the role of these interactions during infection of mosquitoes remains to be assessed.

NsP3 consists of three domains: an N-terminal, conserved macro domain, which possesses phosphatase and RNA-binding activity [24], a conserved zinc-binding central domain [25], and a highly phosphorylated [15,26,27], intrinsically disordered [23], hypervariable domain (HVD) at the C-terminus [6]. Despite the lack of conservation in the HVD between the nsP3 of alphaviruses [28], it contains several conserved motifs, of which the Proline-rich (P-rich) PxxPPR or PxPxPR, and the duplicate FGDF motifs have been studied most extensively (reviewed in [9,10]). In mammalian cells, the P-rich motifs of Sindbis virus (SINV), Semliki Forest virus (SFV) and CHIKV nsP3 interact with the SH3 domain of the membrane modulating proteins amphiphysin-1/2, and this interaction has been shown to be important for efficient virus replication [18,19]. CHIKV nsP3 has two FGDF motifs that can bind to the NTF2-like domain of the stress granule components G3BP1 and G3BP2 in mammalian cells [17,20,29]. This interaction inhibits the formation of cellular stress granules that would otherwise stall host and viral translation, and as a consequence may facilitate virus replication [13,21,30]. In mammalian cells, mutations in the alphavirus nsP3 HVD that disrupt both of the FGDF motifs result in complete inactivation of CHIKV and also attenuates SFV and SINV [17,20,21,31]. Furthermore, deletion of a single FGDF motif in CHIKV or SFV nsP3 decreases the affinity for G3BP and attenuates the virus, suggesting that alphaviruses require duplicate FGDF motifs for their successful replication in mammalian cells [17,20].

While the importance of the conserved motifs in the nsP3 HVD for alphavirus replication in mammalian cells has been well-established, little is known about their role during alphavirus transmission by the mosquito vector [32]. Recently, nsP3 and its mosquito interaction partner Rasputin (Rin), the mosquito homologue of G3BP, have been identified as important determinants for alphavirus transmission [33,34]. Chimeric CHIKV equipped with the nsP3 from the *Anopheles-*transmitted alphavirus ONNV showed an increase in infection rate in the CHIKV-refractory vector *An*. *gambiae* from 0 to 64% [33]. Interestingly, CHIKV chimeras that contained just the C-terminal region of ONNV nsP3, including the HVD, already reached an infection rate of 9–18% [33], suggesting that the nsP3 HVD is an important determinant of mosquito vector specificity. Furthermore, CHIKV nsP3 was shown to co-localize with Rin [34], and Rin co-precipitated with SINV nsP3 [14], indicative of a direct interaction between Rin and nsP3. Interestingly, mutating both FGDF motifs in CHIKV nsP3 together results in complete loss of co-localization of transiently expressed nsP3 and Rin [34], similar to the interaction with G3BP in mammalian cells. However, co-localisation was maintained when only one of the FDGF motifs was mutated [34]. Importantly, knock-down of Rin severely decreased the infection and transmission rates as well as the viral titers of CHIKV in the heads of *Ae*. *albopictus* after an infectious bloodmeal [34]. Thus, the conserved motifs in the nsP3 HVD may play a crucial role in infection of the mosquito vector, potentially through their interaction with mosquito host proteins, and ultimately enable the virus to establish a disseminated infection.

Here, we investigated the role of the P-rich and FGDF motifs in the HVD of CHIKV nsP3 during virus replication in mosquito cells and infection of and transmission by in the natural CHIKV vector *Ae*. *aegypti*. Infectious cDNA clones were used to generate CHIKV variants with mutations in either the P-rich or FGDF motif(s). The mutant viruses were assessed for replication kinetics in mammalian (Vero) and *Ae*. *aegypti* (Aag2) cells. We investigated the infection and transmission of the CHIKV mutants in *Ae*. *aegypti* mosquitoes through infectious bloodmeal experiments. These experiments elucidate whether the evolutionary basis for conservation of the P-rich and duplicate FGDF motifs in alphavirus nsP3 lies solely in infection of the mammalian host, or whether these motifs are also required for alphavirus transmission by the mosquito vector.

## Materials and methods

### Cell culture

African green monkey kidney Vero E6 cells (ATCC CRl-1586) were cultured at 37°C with 5% CO_2_ in Dulbecco’s Modified Eagle medium (DMEM; Gibco) supplemented with 10% fetal bovine serum (FBS; Gibco), penicillin (100 U/ml; Sigma-Aldrich) and streptomycin (100 μg/ml; Sigma-Aldrich). *Ae*. *albopictus* C6/36 (ATCC CRL-1660) cells were cultured at 28°C in Leibovitz L-15 medium (Gibco) supplemented with 10% FBS, 2% tryptose phosphate broth (Gibco) and 1% nonessential amino acids (Gibco). *Ae*. *aegypti* Aag2 cells were cultured at 28°C in Schneider’s *Drosophila* medium (Lonza) supplemented with 10% FBS. During experiments gentamicin (50 μg/ml; Life technologies) was added to the culture medium of C6/36 and Aag2 cells.

### Recombinant viruses

**Fig 1:**
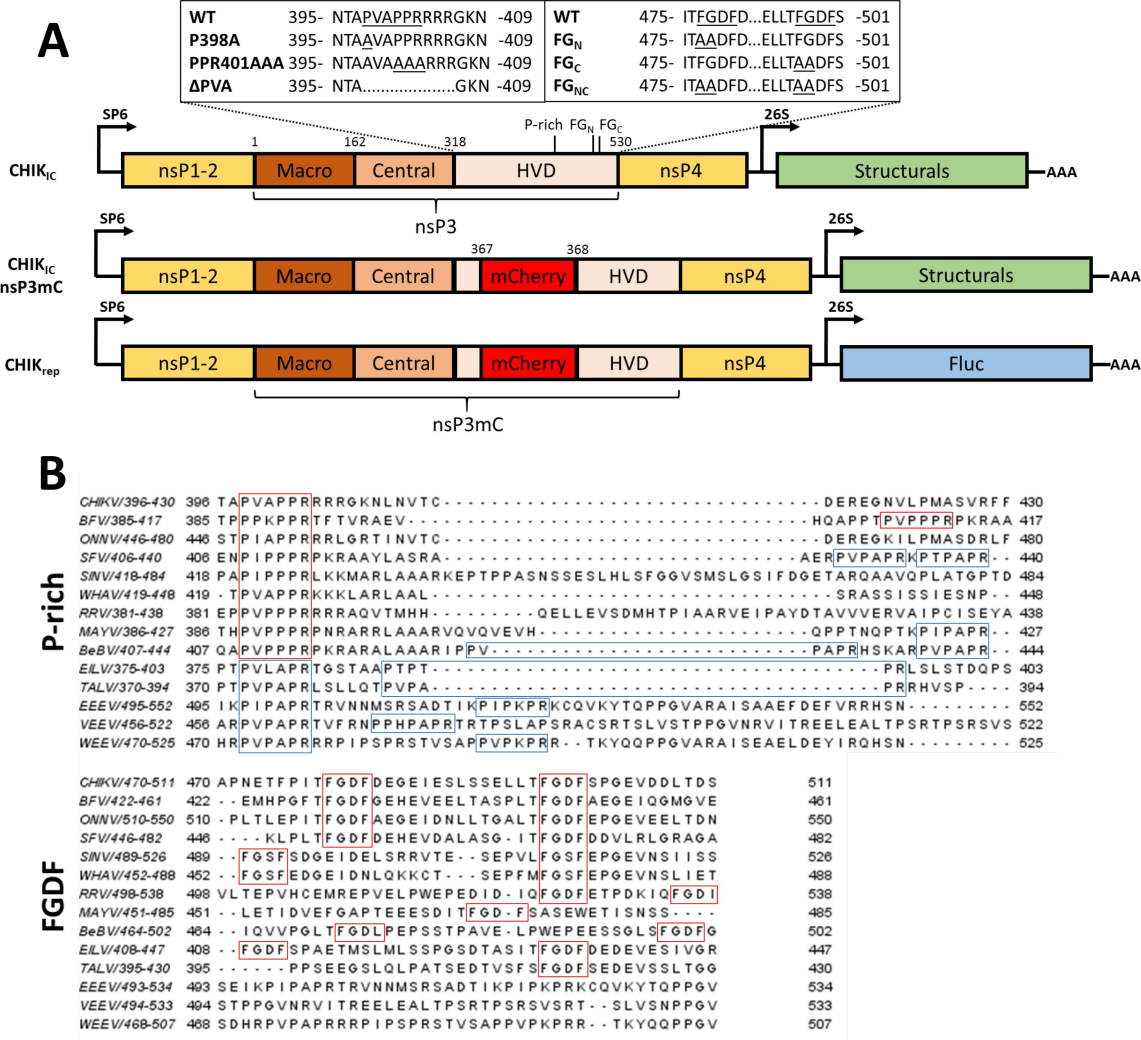
The hypervariable domain of alphavirus nsP3 contains conserved P-rich and FGDF motifs. (A) Schematic overview of the viruses used in these experiments. Shown are DNA plasmids used as template for the *in vitro* transcription of chikungunya virus infectious clone (IC) or replicon (rep) RNA. Additionally, the location of deletions and amino acid substitutions used in mutant IC and rep constructs is displayed. (B) Alignment of alphavirus nsP3 amino acid sequences indicates conserved proline rich and FGDF motifs in the HVD. Top: zoom-in on the proline rich region of the nsP3 HVD. Red boxes indicate the PxxPPR motif, while blue boxes indicate the PxPxPR motif. Bottom: Zoom-in on the FGDF motif-containing region of the nsP3 HVD. CHIKV chikungunya virus; BFV Barmah forest virus; SFV Semliki Forest virus; WHAV Whataroa virus; EILV Eilat virus; RRV Ross River virus; MAYV Mayaro virus; BeBV Bebaru virus; TALV Taï Forest alphavirus; EEEV Eastern equine encephalitis virus; VEEV Venezuelan equine encephalitis virus; WEEV Western equine encephalitis virus.

Previously reported infectious clone derived chikungunya virus 37997 strain (CHIKV^37997^) [35] was used in all experiments. Gateway-compatible pIB vectors with CHIKV nsP3 sequences containing the mutations P398A, PPR401AAA, FG479AA, FG497AA and FG479AA/FG497AA were described previously [34]. To insert an internal fluorescent reporter gene into nsP3, mCherry was PCR amplified from CHIKrep-nsP3mC [13] with primers 1,2 (Primer sequences are listed in Table 1) containing BspEI overhangs and cloned as BspEI fragment into the pIB-nsP3 vectors. This introduces the mCherry sequence between amino acids 367–368 of the CHIKV nsP3 gene. The Firefly luciferase gene was amplified from the previously described pCHIK_rep_-FlucEGFP [13] with primers 7,8 and used to replace the FlucEGFP sequence in the same pCHIK_rep_-FlucEGFP to generate pCHIK_rep_. Mutant nsP3 containing mutations P398A and PPR401AAA in the P-rich motif were cloned from the corresponding pIB vectors as AgeI/NheI fragment into pCHIK_rep_-Fluc to create pCHIK_rep_-nsP3mC^P398A^ and pCHIK_rep_-nsP3mC^PPR401AAA^. NsP3 containing mutations in the FGDF motifs were PCR amplified using primers 3,4, nsP4 was PCR amplified using primers 5,6 and the two PCR products were fused by overlapping-PCR using primers 3,6. Subsequently, the nsP3-nsP4 fusion PCR products were cloned as AgeI/AscI fragment into pCHIK_rep_-Fluc to create pCHIK_rep_-nsP3mC-FG_N_ (FG479AA), pCHIK_rep_-nsP3mC-FG_C_ (FG497AA) and pCHIK_rep_-nsP3mC-FG_NC_ (FG479AA & FG497AA). The Fluc sequence was replaced with the CHIKV^37997^ structural cassette from pCHIK_IC_ through AscI/EcoRI cloning to generate pCHIK_IC_-nsP3mC infectious clones of all mutants. The internal mCherry was removed by BspEI digestion and vector self-ligation. An overview of the recombinant CHIKV encoding plasmids used in this study is shown in Fig 1A. Recombinant viruses were generated from the pCHIK_IC_ infectious clones as described previously [35]. Briefly, pCHIK_IC_ plasmids were linearized by PacI digestion to serve as template for an SP6 RNA polymerase (NEB) transcription reaction and the resulting *in vitro* transcribed RNA was used to transfect pre-seeded Vero cell monolayers with Lipofectamine 2000 (Invitrogen) to generate the P0 stock. Subsequent passages were performed on C6/36 cells.

**Table 1:** Primers used in this study.

#	Name	Sequence (5’ —> 3’)
1	mCherry-F	ATTCCGGAAATGGTGAGCAAGGGCGAG
2	mCherry-R	GTTCCGGAATCTTGTACAGCTCGTCCATG
3	nsP3-F	TAAACAGCGTAGCTATACCG
4	nsP3_end-R	CCCACCTGCCCTATCTAGTC
5	nsP3_end-F	GACACGGACGACGAATTATG
6	nsP4-R	TTCCATGGCGCGCCGTAG
7	Fluc-F	GGCCACCTTTGCAAGCTCT
8	Fluc-R	GAATTCCTCCACGGCGATCTTTCCG
9	pPUB-F	AATCATGAATCTTTACATGTAGCTTGTGCA
10	pPUB-R	AAGAGCTCGGTTGAAATCTCTGTTGAGCA
11	pPUB-EcoRI-F	GGACCGGTATGGTGAGCAAGGGCGAGG
12	pPUB-EcoRI-R	GGGAGCTCCCTTGTACAGCTCGTCCATGCC
13	GFP-KpnI-F	GGGGGGTACCATGGTGAGCAAGGGCGAGGAG
14	GFP-SacII-R	GGGGCCGCGGCTACTTGTACAGCTCGTCCATGCCGAG

### Expression plasmids

Gateway-compatible and OpIE-2 promoter driven pIB insect-cell expression plasmids expressing EGFP, nsP3EGFP, nsP3EGFP-FG_N_, nsP3EGFP-FG_C_ and nsP3EGFP-FG_NC_ were reported previously [34]. The *Ae*. *aegypti* poly-ubiquitin (PUB) promoter was amplified from pPUB-Fluc [36] using primers 9,10 (Table 1) containing BspHI and SacI restriction sites and cloned as BspHI/SacI fragment into pIB to generate the Gateway-compatible pPUB vector. Gateway-cloning (Invitrogen) was used to generate pPUB-EGFP, pPUB-nsP3EGFP, pPUB-nsP3EGFP-FG_N_, pPUB-nsP3EGFP-FG_C_ and pPUB-nsP3EGFP-FG_NC_. Plasmid pIB-Rin-mC, expressing the mosquito Rin fused C-terminally to an mCherry red fluorescent reporter gene, was reported previously [34]. EGFP was PCR amplified from pEGFP-N1 with primers 13,14 (Table 1) containing KpnI and SacII overhangs and cloned as KpnI/SacII fragment into pIB-Rin-mC to generate pIB-Rin-EGFP. The PUB-promoter was amplified from pPUB-EGFP with primers 11,12 (Table 1) containing EcoRI overhangs and cloned as EcoRI/EcoRI fragment into pIB-Rin-mC and pIB-Rin-EGFP to generate pPUB-Rin-mC and pPUB-Rin-EGFP respectively.

### Virus growth curves

Vero or Aag2 cell monolayers were seeded one day prior to infection in a 6-wells plate. The amount of inoculum for the specific multiplicity of infection (MOI) was calculated based on end-point dilution assay (EPDA) on either Vero or Aag2 cells. Cell monolayers were infected with 1 ml of inoculum in either DMEM- 4-(2-hydroxyethyl)-1-piperazineethanesulfonic acid (HEPES) (Gibco) supplemented with 10% FBS, streptomycin and penicillin (DMEM-supplemented) or Schneider’s *Drosophila* medium supplemented with 10% FBS and gentamycin (50 μg/ml) (Schneider’s-supplemented). After 2 h the inoculum was aspirated, monolayers were washed with phosphate-buffered saline (PBS) and 2 ml of fresh culture medium was added. Vero cells were incubated at 37°C with 5% CO_2_ and Aag2 cells were incubated at 28°C. At the indicated time points post infection 30 μl supernatant samples were taken and after one freeze-thaw cycle (-80°C) the infectious virus titre was determined by EPDA on either Vero or Aag2 cells.

### End-point dilution assays

Vero cells were detached by incubation with Trypsin-EDTA (Gibco) and diluted 1:4 in DMEM-supplemented, while Aag2 cells were detached by force and diluted 1:8 with Schneider’s supplemented. Samples were thawed, vortexed and 10-fold serial dilutions were made in either DMEM-supplemented or Schneider’s-supplemented. Cell suspensions were added to the dilutions 1:1 and 10 μl of inoculated dilution was plated 6-fold in micro-titer plates (Nunc). When samples from mosquitoes were titrated, Fungizone (50 μg/ml; Invitrogen) and gentamycin (50 μg/ml; Life Technologies), was added to the medium and cell fractions prior to titrations (DMEM-complete, Schneider’s-complete). Titrations on Vero cells were scored by CHIKV-induced CPE, while titrations on Aag2 cells were scored by α-CHIKV-E2 based immunofluorescence assay.

### Immunofluorescence assay

Vero or Aag2 cells were fixed by >10 min incubation in 4% paraformaldehyde in PBS. Cells were permeabilized by >10 min incubation in 0.1% sodium dodecyl sulphate in PBS, washed three times with PBS and blocked by incubation in 5% FBS in PBS for 30 min. Primary antibody binding was performed with α-CHIKV-E2 (rabbit polyclonal; 1:5000 [37]) or α-G3BP1 (1:500; G6046 Sigma Aldrich) diluted in 5% FBS in PBS for 1 h at room temperature (RT). Cells were washed three times with PBS and incubated with the secondary antibody goat-α-rabbit-Alexa Fluor 488 (1:1000; Invitrogen) in 5% FBS in PBS for 1 h at 37°C. Cells were washed three times with PBS and visualized using an Axio Observer Z1m inverted microscope (Zeiss) in combination with an X-Cite 120 series lamp.

### Infectious blood meal experiments

*Ae*. *aegypti* mosquitoes (Rockefeller strain, obtained from Bayer AG,) were reared as described previously [35] and transported to the Biological Safety level 3 facility in buckets (diameter: 12.2 cm, height: 12.2 cm; Jokey) for virus infection assays. Mosquitoes were starved for one day and then offered an infectious bloodmeal from a 1:1 mixture of virus and human blood (Sanquin) in a total volume of 1 ml via a Hemotek feeder covered with a Parafilm membrane. Mosquitoes were allowed to feed for 1 h under light conditions at 24°C and 70% relative humidity (RH). Mosquitoes were anesthetized with CO_2_ and fully engorged females were selected and maintained in buckets at 28°C, 12:12 light:dark cycle, 70% relative humidity and provided with a cotton pad soaked in 6% glucose solution. After 7 or 14 days, mosquitoes were anesthetized with 100% CO_2_, and immobilized by removing their legs and wings. The proboscis of each stripped mosquito was inserted into a 200 μl pipet tip containing 5 μl of a 1:1 mixture of 50% glucose and FBS. Mosquitoes were allowed to salivate for >45 min, after which the bodies were individually transferred to 1.5 ml Safe-Seal micro tubes (Sarstedt) that contained a small scoop of 0.5 mm zirconium beads (Next Advance). Saliva samples were added to 1.5 ml micro tubes (Sarstedt) that contained 60 μl DMEM-complete. All samples were stored at -80°C until further processing. For most experiments, transmission was determined at 7 days post infection (dpi), as wild type CHIKV reaches relatively high transmission rates at this time-point. This therefore presents a good resolution for the detection of a putative attenuation of a mutant virus.

### Infectivity assay

Frozen mosquito bodies were homogenized for 2 min at maximum speed in a Bullet Blender Storm (Next Advance). Homogenized bodies were centrifuged for 1 min at 14.500 rpm, after which 100 μl of DMEM-complete was added and the homogenization was repeated. Finally the mosquito debris was pelleted by 1 min centrifugation at 14.500 rpm. Thirty microliters of mosquito body supernatant or saliva samples were used to inoculate either Vero or Aag2 cell monolayers in 96-wells plates. After 2–3 h incubation the inoculum was removed and replaced with fresh DMEM-complete or Schneider’s-complete. Infectivity was scored at 3 dpi through virus-induced CPE in Vero cells or by CHIKV-E2-based immunofluorescence in Aag2 cells, as described above.

### Verification of nsP3 mutants

Mosquitoes with CHIKV-positive saliva were selected from mosquitoes inoculated with CHIK_IC_, CHIK_IC_^P398A^, CHIK_IC_^PPR401AAA^, CHIK_IC_-FG_N_ or CHIK_IC_-FG_C_. A selection of mosquitoes was made to represent each replicate experiment. Thirty μl of mosquito sample supernatant was used to inoculate a well of a 96-wells plate pre-seeded with C6/36 cells. At 3 days post infection RNA was isolated with TRIzol reagent (Invitrogen) and 300 ng total RNA was subjected to one-step RT-PCR with primers 3,4 using SuperScript III one-step RT-PCR system with Platinum Taq DNA polymerase (Invitrogen) following the manufacturer’s protocol. RT-PCR products were observed on agarose gel to confirm the infection of mosquitoes with CHIKV. To investigate the presence and preservation of the expected mutations, RT-PCR products were subjected to digestion with SacII (NEB), NotI (NEB) or ApeKI (NEB) according to the manufacturer’s protocol. Digestions were observed on agarose gel.

### Transfections

Transfections of mosquito cells with DNA plasmids were performed with Fugene-HD (Promega) in serum-free media following the manufacturer’s protocol. Transfections of Vero cells with DNA plasmids and transfections of C6/36, Aag2 and Vero cells with *in vitro* transcribed RNA were performed using Lipofectamine 2000 (Invitrogen) in Opti-MEM (Gibco). At 4 hours post transfection (hpt) with Lipofectamine 2000 the transfection mix was replaced with fresh culture media.

### Luciferase measurements

The cell culture volume was aspirated and cells were lysed by 20 min incubation in 1X passive lysis buffer (Promega) at RT. Ninety μl of cell lysate was pipetted into an opaque 96-wells plate and 90 μl of Steady-Glo reagent (Promega) was added. After 10 min the luciferase activity was measured using a FLUOstar Optima microplate reader (BMG Labtech).

### Co-immunoprecipitation and western blotting

Pre-seeded Vero or Aag2 cells were transfected with plasmids expressing nsP3 or Rin or both. One day post-transfection cells were detached, washed once with PBS and lysed by 30 min incubation in lysis buffer (10 mM Tris/Cl pH 7.5; 150 mM NaCl; 0.5 mM EDTA; 0.5% NP-40) supplemented with Complete protease inhibitors (Roche) and 1 mM phenylmethylsulfonyl fluoride (PMSF). Lysates were cleared by 10 min 20.000 g centrifugation at 4°C and diluted 5:2 with dilution buffer (10 mM Tris/Cl pH 7.5; 150 mM NaCl; 0.5 mM EDTA). Twenty-five μl GFP-Trap_A beads (Chromotek) were added to 500 μl dilution buffer and pelleted by 2 min 2,500 g centrifugation at 4°C. Washing was repeated twice. Cell lysates were added to the equilibrated beads and incubated for 1–2 h at 4°C with overhead rotation. Beads were washed thrice by resuspension in dilution buffer followed by 2 min 2,500 g centrifugation at 4°C. Bound protein complexes were eluted by resuspension in 2X SDS-loading buffer containing 10% β-mercaptoethanol followed by 10 min incubation at 95°C. Beads were removed by 5 min 3,000 g centrifugation and eluates were subjected to western blotting. Briefly, proteins were size-separated by SDS-PAGE and semi-dry blotted onto Immobilon-P membranes (Merck Millipore). Membranes were blocked overnight at 4°C by incubation in 1% milk powder/PBS-0.05%-Tween-20 (PBST). Blocked membranes were probed for 1 h at RT with primary antibodies α-G3BP (1:1000; G6046 Sigma Aldrich), α-GFP (1:2000; A6455 Molecular Probes), α-mCherry (1:1000; ab183628 Abcam) diluted in 1% milk powder/PBST. Membranes were washed thrice for 5 min with PBST and probed for 1 h at RT with alkaline phosphatase (AP) conjugated secondary antibody goat-α-rabbit-AP (1:2500; D0487 Dako) diluted in PBST. Membranes were washed thrice for 5 min in PBS-T and developed with nitroblue tetrazolium (NBT)/BCIP (5-bromo-4-chloro-3-indolylphosphate) (Roche) until the desired signal was achieved.

### Statistics

Statistical analysis were performed in SPSS Statistics 23. Virus titers in mosquito bodies and supernatants from growth curve experiments were checked for normality by Kolmogorov-Smirnov test. Data that did not follow a normal distribution was Log_10_ transformed and confirmed for normality. Means were compared by one-way ANOVA with Tukey post-hoc test (α = 0.05). Data that did not follow a normal distribution, despite Log_10_ transformation was analysed by Kruskal-Wallis test with Dunn’s post-hoc test (α = 0.05). Two-tailed Fisher’s exact tests were used to compare infection and transmission rates and performed with GraphPad QuickCalcs (α = 0.05). Ns non-significant; * *P* < 0.05; ** *P* ≤0.01; *** *P* ≤0.001.

## Results

### The CHIKV nsP3 P-rich motif is required for efficient virus replication in mammalian and mosquito cells

**Fig 2:**
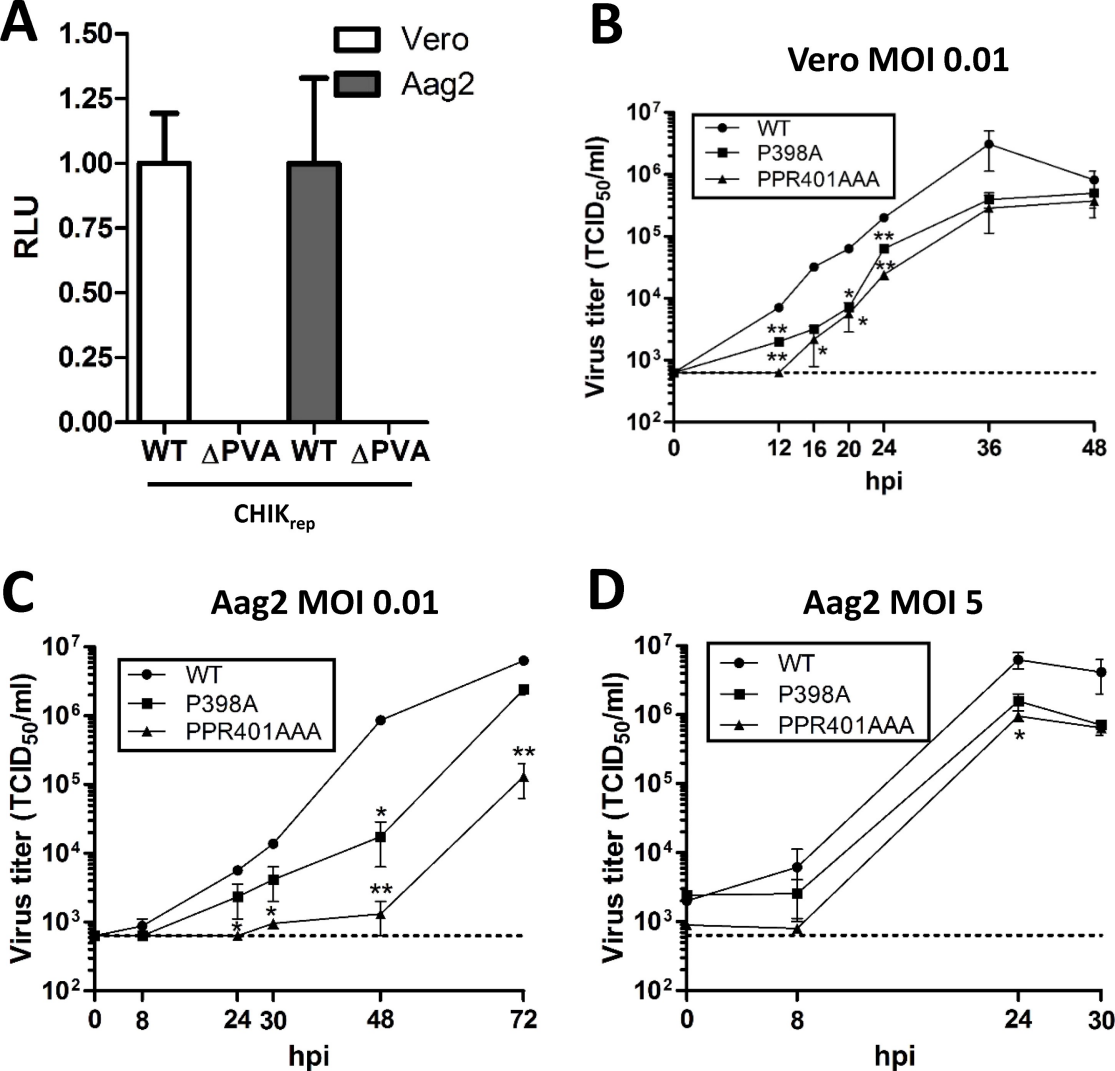
The P-rich motif is important but not essential for chikungunya virus replication in mammalian and mosquito cells. (A) Vero and Aag2 cells were transfected with *in vitro* transcribed RNA of CHIK_rep_ or CHIK_rep_^ΔPVA^ and the relative luciferase expression was quantified at 24 hpt. Bars indicate the mean relative light units (RLU) ±standard error of the mean from three independent experiments. (B/C) Vero (B) and Aag2 (C) cells were infected in duplicate with CHIK_IC_, CHIK_IC_^P398A^ or CHIK_IC_^PPR401AAA^ at a multiplicity of infection (MOI) of 0.01. TCID_50_/ml was determined by EPDA on Vero cells at the indicated time-points. (D) Aag2 cells were infected in duplicate with CHIK_IC_, CHIK_IC_^P398A^ or CHIK_IC_^PPR401AAA^ at an MOI of 5 and the TCID_50_/ml was determined by EPDA on Vero cells at the indicated time-points. Asterisks indicate significant differences compared to the wild type virus by one-way ANOVA with Tukey’s post-hoc test on Log_10_ transformed data at each time-point (α = 0.05). The dotted line in panels B-D indicates the EPDA detection limit.

For SINV, SFV and CHIKV it has been shown that deletion or mutation of the conserved P-rich motif (Fig 1B) results in decreased replication in mammalian cells [13,18]. To investigate whether the P-rich motif is similarly indispensable for alphavirus replication in mosquito cells luciferase assays were performed on cells transfected with *in vitro* transcribed RNA of a CHIKV replicon (CHIK_rep_) with an in-frame deletion of the P-rich motif (ΔPVA) (Fig 1A, Fig 2A). The mutant replicon CHIK_rep_^ΔPVA^ was unable to replicate in either mammalian Vero or Aag2 *Ae*. *aegypti* mosquito cells, whereas wild type CHIK_rep_ was able to replicate in both cell types. We also transfected cells with a CHIKV infectious clone containing the same deletion of the P-rich motif (CHIK_IC_^ΔPVA^), but again we were unable to produce infectious virus in either mammalian and mosquito cells. Together, these results clearly indicate that the P-rich motif is essential for CHIKV replication in both mammalian and mosquito cells.

To investigate whether the entire, intact P-rich motif is required for virus production in mammalian or mosquito cells, amino acid substitutions were made in the CHIKV infectious clone (CHIK_IC_) to produce CHIK_IC_^P398A^ and CHIK_IC_^PPR401AAA^ (Fig 1A). These specific mutations are designed to disrupt the residues in the P-rich motif that are conserved among old-world alphaviruses (PxxPPR; Fig 1B red boxes). Viral growth curves were determined in duplicate using an MOI of 0.01 and viral titers were determined by EPDA on Vero cells. The results show that both mutants displayed significantly delayed growth kinetics compared to the wild type virus in both Vero (Fig 2B) and Aag2 cells (Fig 2C). Even when virus infections were performed at a higher MOI of 5 this attenuating effect was observed at 24 hpi, although both CHIK_IC_^P398A^ and CHIK_IC_^PPR401AAA^ still reached a titer of ~1.0 × 10^6^ TCID_50_/ml (Fig 2D). Together, these results indicate that an intact P-rich motif in CHIKV nsP3 is important, but not essential, for virus replication in mammalian and mosquito cells.

### The P-rich motif is not required for the transmission of chikungunya virus by *Ae*. *aegypti* mosquitoes

**Fig 3:**
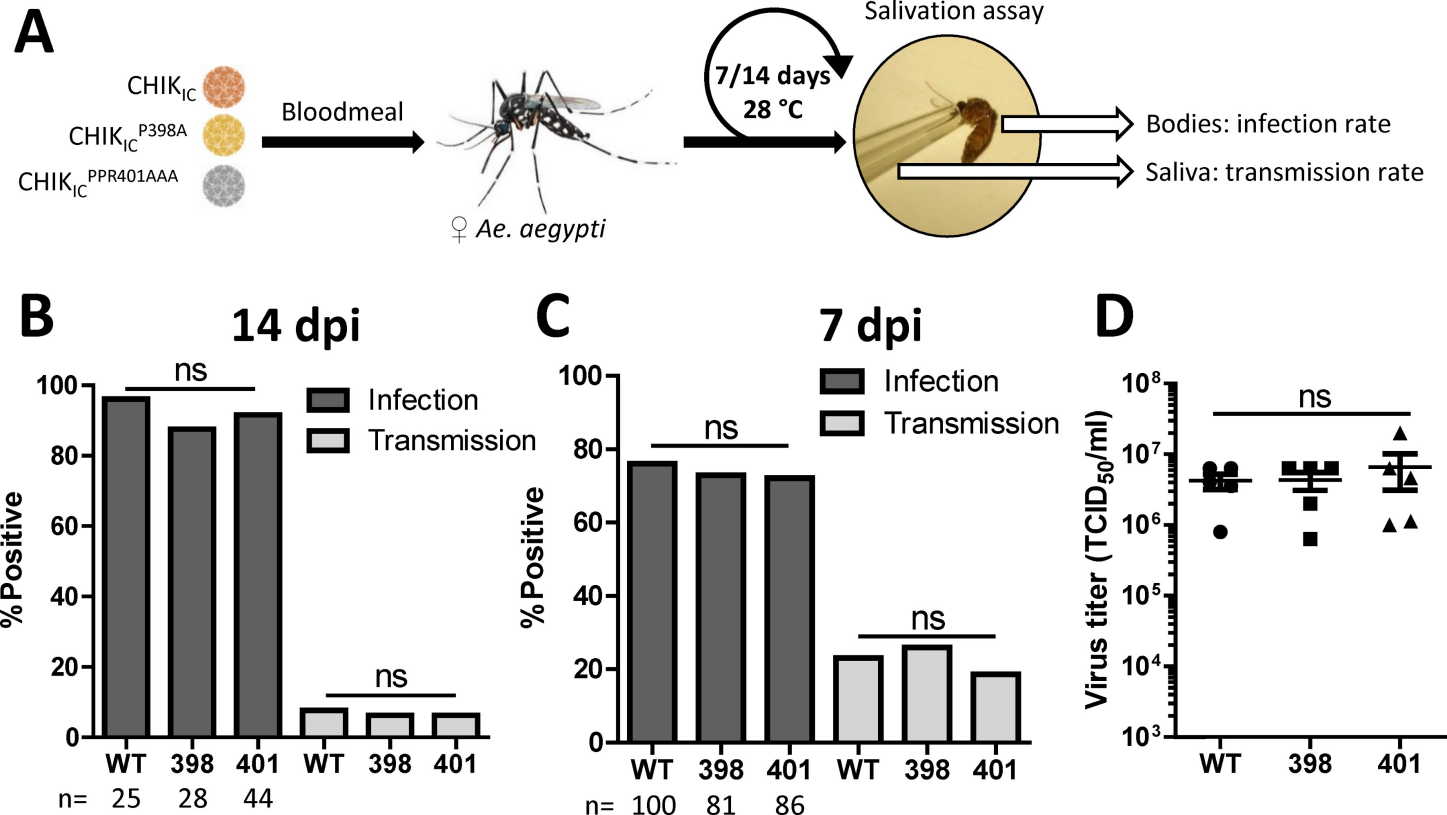
The P-rich motif is not required for transmission of chikungunya virus by *Aedes aegypti* mosquitoes. (A) Schematic experimental set-up. Female *Ae*. *aegypti* mosquitoes were infected through an infectious bloodmeal containing 1.0 × 10^7^ TCID_50_/ml of CHIK_IC_, CHIK_IC_^P398A^ or CHIK_IC_^PPR401AAA^_._ At 14 days post infection (dpi) (B) or 7 dpi (C) the infection and transmission rates were determined by infectivity assay on Vero cells. Bars represent cumulative numbers from three (B) or two (C) independent experiments. n = total number of mosquitoes used per treatment. Statistics were performed by Fisher’s exact test (α = 0.05). (D) TCID_50_/ml of CHIKV in the bodies *Ae*. *aegypti* mosquitoes with CHIKV-positive saliva at 7 dpi were determined by end-point dilution assay on Vero cells. Statistics were performed by one-way ANOVA with Tukey’s post-hoc test on Log_10_ transformed data (α = 0.05).

The P-rich motif is conserved in most if not all mosquito transmitted alphaviruses and also in the insect-specific alphaviruses Tai Forest Alphavirus (TALV) and Eilat virus (EILV) (Fig 1B). As TALV and EILV are proposedly unable to replicate in a vertebrate host [38,39], conservation of a P-rich motif for these viruses suggests its importance for alphavirus infection in the invertebrate vector. To investigate whether the P-rich motif of CHIKV nsP3 is involved in virus transmission by mosquitoes, female *Ae*. *aegypti* were offered an infectious bloodmeal containing either CHIK_IC_, CHIK_IC_^P398A^ or CHIK_IC_^PPR401AAA^ (Fig 3A). After 14 days, the infection and transmission rates were determined by infectivity assay on Vero cells (Fig 3B). For all three viruses, similar infection (88–96%; *P >* 0.37) and transmission (6–7.7%; *P =* 1.00) rates were obtained, indicating that the P-rich motif is not required for virus transmission by mosquitoes.

To investigate whether putative differences could be observed earlier after infection of the mosquito vector, we also determined the infection and transmission rates after 7 days (Fig 3C). Again, infection (72–76%; *P* > 0.61) and transmission (18–23%; *P* > 0.47) rates were obtained that were not significantly different when compared to the wild type virus. In order to determine whether the mutations in CHIK_IC_^P398A^ and CHIK_IC_^PPR401AAA^ were preserved during infection of the mosquito, the nsP3 sequences were amplified by RT-PCR for a selection of mosquitoes with CHIKV-positive saliva. Presence of the expected mutations in nsP3 was confirmed, indicating that the mutant viruses did not revert to wild type during replication in the mosquito vector (S1 Fig). To assess whether the titers in the mosquito bodies would perhaps be decreased due to the mutations in the P-rich motif, CHIKV titers were determined in the bodies of mosquitoes with virus-positive saliva (Fig 3D). However, no difference in viral titer between CHIK_IC_, CHIK_IC_^P398A^ and CHIK_IC_^PPR401AAA^ (*P* > 0.99) was observed. Together, these results indicate that the CHIKV nsP3 P-rich motif is not required for the transmission by *Ae*. *aegypti* mosquitoes.

### Chikungunya virus nsP3 requires duplicate FGDF motifs for replication in mammalian cells and a single FGDF motif in mosquito cells

**Fig 4:**
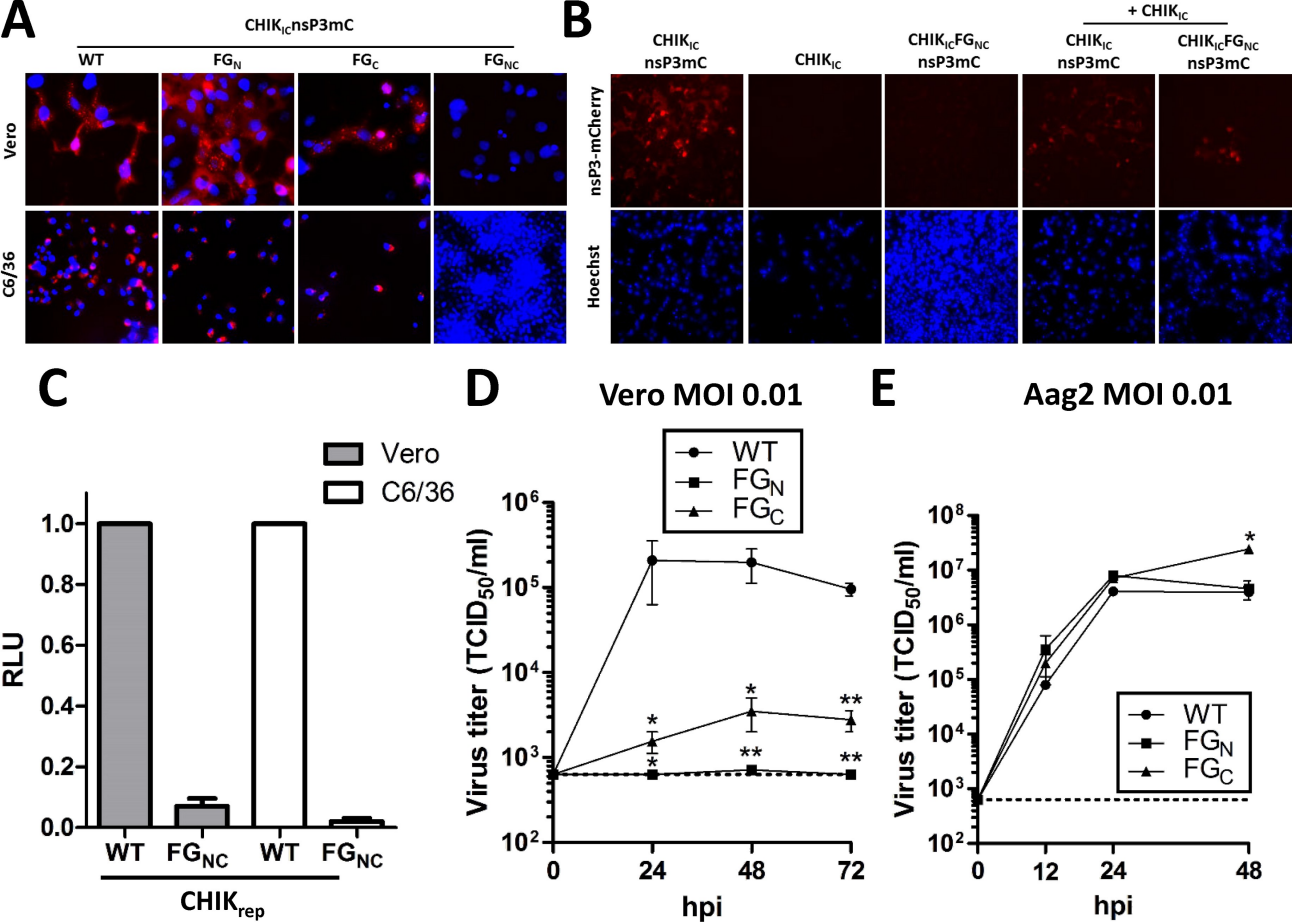
At least one FGDF motif is required for chikungunya virus replication in mammalian and mosquito cells. (A) Vero and C6/36 cells were transfected with *in vitro* transcribed RNA of CHIK_IC_nsP3mC, CHIK_IC_nsP3mC-FG_N_, CHIK_IC_nsP3mC-FG_C_, or CHIK_IC_nsP3mC-FG_NC_. Cells were fixed at 36 hours post transfection, stained with Hoechst, and fluorescence was observed by fluorescence microscopy. (B) Vero cells were transfected with *in vitro* transcribed RNA of CHIK_IC_nsP3mC or CHIK_IC_nsP3-FG_NC_ either individually or co-transfected with *in vitro* transcribed RNA of CHIK_IC_. Cells were fixed at 36 hpt, stained with Hoechst and fluorescence was observed by fluorescence microscopy. (C) Vero and C6/36 cells were transfected with *in vitro* transcribed RNA of CHIK_rep_ or CHIK_rep_-FG_NC_ and the relative luciferase expression was quantified at 24 hpt. Bars indicate the mean relative light units (RLU) ±SEM, normalized to the wild type replicon from at least three independent experiments. (D) Growth curves of CHIKV_IC_, CHIKV_IC_-FG_N_ and CHIKV_IC_-FG_C_ on Vero cells infected in duplicate with an MOI of 0.01 based on end-point dilution assay (EPDA) on Vero cells. At the indicated time-points the TCID_50_/ml was determined by EPDA on Vero cells. (E) Growth curves of CHIKV_IC_, CHIKV_IC_-FG_N_ and CHIKV_IC_-FG_C_ on Aag2 cells infected in duplicate with an MOI of 0.01 based on infectivity on Aag2 cells. At the indicated time-points the TCID_50_/ml was determined by EPDA on Aag2 cells. Statistics were performed by one-way ANOVA with Tukey’s post-hoc test on Log_10_ transformed data at each time-point (α = 0.05). Asterisks indicate significance compared to the wild type virus. The dotted line in panels D-E indicates the EPDA detection limit.

Research has indicated that mutating both FGDF motifs together significantly attenuates SFV and renders CHIKV unable to replicate in mammalian cells [17,20]. Here, we investigated whether mutations in one (nsP3-FG_N_, nsP3-FG_C_) or both (nsP3-FG_NC_) FGDF motifs would interfere with the replication of CHIKV in mosquito cells. These amino acid mutations were previously shown to disrupt the interaction of nsP3 with G3BP [20,40], and the co-localization of nsP3 with Rin [34]. We tested the effect of mutating the FGDF motifs on CHIKV replication using an infectious clone that contains an in-frame insertion of the mCherry reporter gene in nsP3 (CHIK_IC_nsP3mC; Fig 1A). *In vitro* transcribed, capped RNA from CHIK_IC_nsP3mC_,_ CHIK_IC_nsP3mC-FG_N_, CHIK_IC_nsP3mC-FG_C_ or CHIK_IC_nsP3mC-FG_NC_ was used to transfect Vero and C6/36 mosquito cells. At 36 hpt, the cells were fixed and analysed for mCherry fluorescence as a marker for virus replication (Fig 4A). Red fluorescence was observed in cells transfected with RNA from the infectious clones with mutations in a single FGDF motif (CHIK_IC_nsP3mC-FG_N_ and CHIK_IC_nsP3mC-FG_C_) and wild type CHIKV. However, mammalian and mosquito cells transfected with RNA from CHIK_IC_nsP3mC-FG_NC_ did not show any red fluorescence, indicating that a minimum of one functional FGDF motif is essential for CHIKV replication in both mammalian and mosquito cells.

To assess whether the RNA of CHIK_IC_nsP3mC-FG_NC_ was of sufficient quality, we co-transfected replication-competent RNA from CHIK_IC_, which does not express mCherry. In this experiment, CHIK_IC_ RNA is able to complement the CHIK_IC_nsP3mC-FG_NC_ RNA, leading to mCherry expression. Indeed, upon co-transfection red fluorescence could be observed in some cells (Fig 4B), indicating that the quality of CHIK_IC_nsP3mC-FG_NC_ RNA was fine but that the RNA was replication-incompetent due to the mutation of both FGDF motifs. To confirm this result using a more sensitive assay, Vero and C6/36 cells were transfected with CHIKV replicons expressing firefly luciferase CHIK_rep_ or CHIK_rep_-FG_NC_ (Fig 1A) and firefly luciferase activity was measured at 24 hpt (Fig 4C). In both Vero and C6/36 cells, only the wild type replicon CHIK_rep_ produced above-background luciferase levels, indicating that indeed mutation of both FGDF motifs together results in complete inactivation of CHIKV in mammalian and mosquito cells.

To assess the effect of single FGDF mutations on virus production in mammalian and mosquito cells, we performed viral growth curves on Vero and Aag2 cells at an MOI of 0.01 using CHIK_IC_, CHIK_IC_-FG_N_ and CHIK_IC_-FG_C_ (Fig 4D and 4E). These viruses do not contain the mCherry reporter gene (Fig 1A). It is important to note that we determined the viral titers for the inoculum and the samples for the mammalian growth curves by EPDA on Vero cells. For the growth curves on mosquito cells, the titrations were performed by EPDA on Aag2 cells. As expected, CHIK_IC_-FG_N_ and CHIK_IC_-FG_C_ were severely attenuated in Vero cells (Fig 4D). However, the result was very different in Aag2 cells; CHIK_IC_-FG_N_ and CHIK_IC_FG_C_ displayed remarkably similar growth rates as compared to CHIK_IC_ (Fig 4E). In summary, duplicate FGDF motifs are very important for CHIKV replication in mammalian cells, while a single FGDF motif is sufficient, but required, for efficient CHIKV replication in mosquito cells.

### Requirement of FGDF motifs for chikungunya virus transmission by *Aedes aegypti*

**Fig 5:**
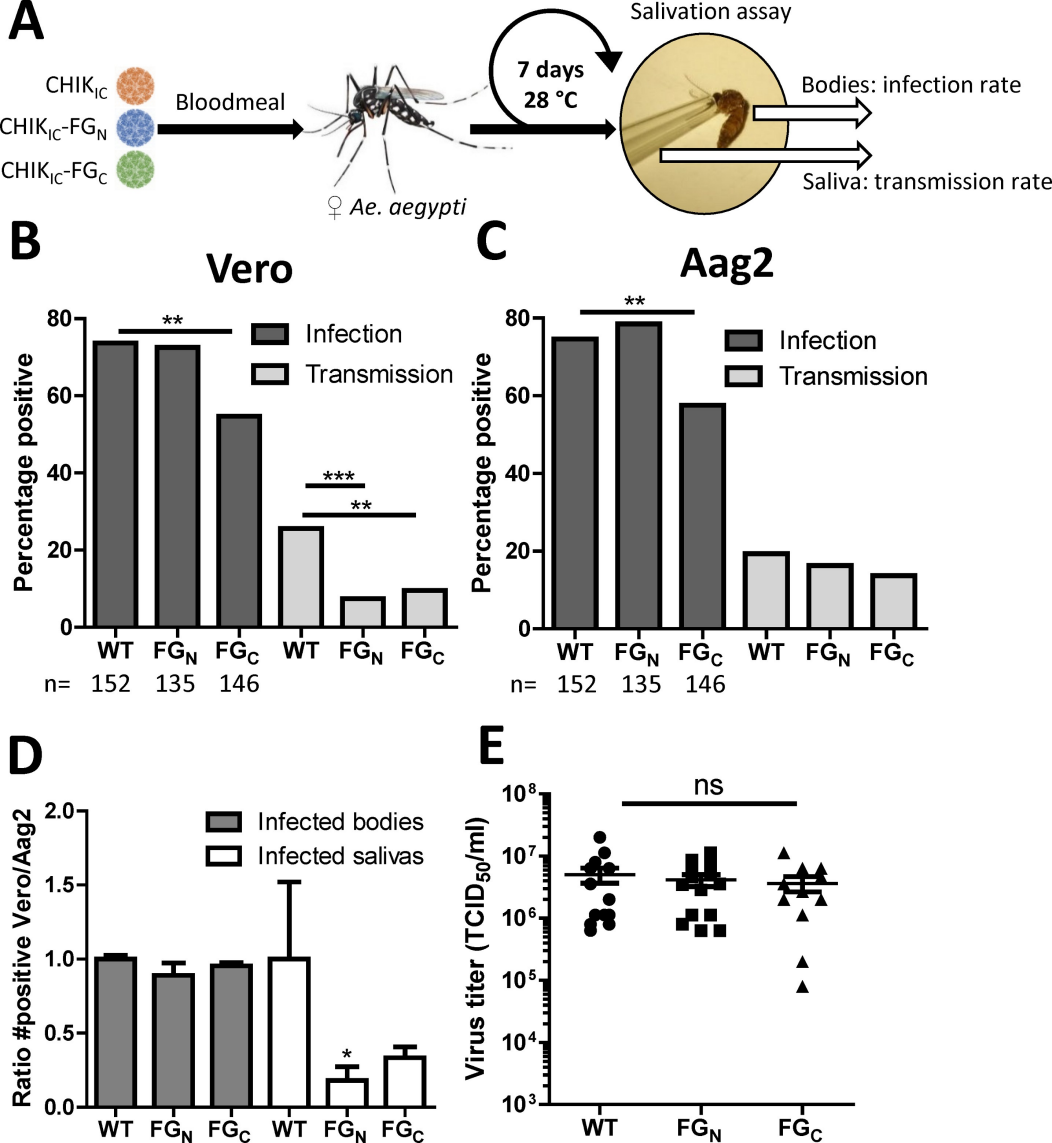
A single FGDF motif is sufficient for the transmission of chikungunya virus by *Aedes aegypti* mosquitoes. (A) Schematic experimental set-up. Female *Ae*. *aegypti* mosquitoes were infected through an infectious bloodmeal containing 2.8 × 10^5^ TCID_50_/ml of CHIK_IC_, CHIK_IC_-FG_N_, CHIK_IC_-FG_C._ At 7 days post infection (7dpi) the infection and transmission rates were determined by infectivity assay on (B) Vero and (C) Aag2 cells. Bars represent cumulative numbers from three independent experiments. n = total number of mosquitoes used per treatment. Statistics were performed by Fisher’s exact test (α = 0.05). (D) Ratio between the number of positive bodies or salivas in Vero compared to Aag2 cells. Statistics were performed by Kruskal-Wallis test with Dunn’s post-hoc test (α = 0.05). (E) TCID_50_/ml in the bodies of *Ae*. *aegypti* mosquitoes with CHIKV-positive saliva at 7 dpi were determined by end-point dilution assay on Aag2 cells. Statistics was performed by one-way ANOVA with Tukey’s post-hoc test on Log_10_ transformed data (α = 0.05).

While duplicate FGDF motifs do not appear to be required for virus replication in mosquito cells (Fig 4E), they might be important to sustain transmission of CHIKV by mosquitoes. Therefore, we infected *Ae*. *aegypti* mosquitoes via an infectious bloodmeal with 2.8 × 10^5^ TCID_50_/ml CHIK_IC_, CHIK_IC_-FG_N_ or CHIK_IC_-FG_C_ and determined the infection and transmission rates at 7 dpi by infectivity assay on Vero and Aag2 cells (Fig 5A). When scored on Vero cells the mosquito infection rate of CHIK_IC_-FG_C_ (54.8%; *P* <0.001) was significantly lower as compared to CHIK_IC_ (73.7%), while the infection rate of CHIK_IC_-FG_N_ (72.6%; *P* = 0.89) was similar to wild type (Fig 5B). Next, the saliva of infected mosquitoes was tested for the presence of virus. This showed that the transmission rates of both CHIK_IC_-FG_N_ (7.4%; *P* <0.001) and CHIK_IC_-FG_C_ (9.6% *P* <0.001) were significantly lower as compared to CHIK_IC_ (25.7%), indicating that duplicate FGDF motifs are important for CHIKV transmission by *Ae*. *aegypti* to the vertebrate host.

However, given that CHIK_IC_-FG_N_ and CHIK_IC_-FG_C_ were severely attenuated on Vero cells (Fig 4D), we also determined the infection and transmission rates by infectivity assay on Aag2 cells, using exactly the same mosquito bodies and salivas (Fig 5C). The infection rate did not change much, and CHIK_IC_-FG_C_ (57%; *P* <0.01) still reached a lower infection rate than CHIK_IC_ (74.7%) or CHIK_IC_-FG_N_ (78.5%; *P =* 0.49). Interestingly, however, when scored on Aag2 cells the transmission rates were very similar for CHIK_IC_ (19.3%), CHIK_IC_-FG_N_ (16.3%; *P* = 0.54) and CHIK_IC_-FG_C_ (13.7%; *P* = 0.21)_._ To directly compare the infectivity of the mosquito bodies and salivas between Vero and Aag2 cells, the ratio between the number of positive samples on Vero and Aag2 cells was determined for each replicate and normalized to the ratio for mosquitoes inoculated with wild type CHIK_IC_ (Fig 5D). Indeed, the ratio of Vero/Aag2 positive samples was lower for CHIK_IC_-FG_N_ and CHIK_IC_-FG_C_ as compared to wild type CHIK_IC_ (FG_N_ 0.18; FG_C_ 0.33). To determine whether the mutations in CHIK_IC_-FG_N_ and CHIK_IC_-FG_C_ were preserved during replication in the mosquito, the nsP3 sequences of a selection of mosquitoes with CHIKV-positive saliva were amplified by RT-PCR. Presence of the expected mutations in nsP3 was confirmed, indicating that the mutant viruses did not revert to wild type (S1 Fig). Together, these results imply that, while CHIK_IC_-FG_N_ and CHIK_IC_-FG_C_ are perfectly able to infect the mosquito body and disseminate to ultimately reach the mosquito saliva, their virions are less able to replicate in vertebrate cells. These results are in agreement with the observed differences between the growth curves on Vero and Aag2 cells (compare Fig 4D with Fig 4E). To assess the effect of mutating one FGDF motif on the viral load in the mosquito body, the viral titers in mosquito bodies of which the saliva was virus positive were determined by EPDA on Aag2 cells (Fig 5E). Viral loads of mosquitoes infected with CHIK_IC_, CHIK_IC_-FG_N_ or CHIK_IC_-FG_C_ were not significantly different (2.7–5.0 × 10^6^ TCID_50_/ml; *P* > 0.65). Thus, a single FGDF motif in nsP3 is sufficient for the infection and dissemination of CHIKV in *Ae*. *aegypti* mosquitoes, but duplicate FGDF motifs are required for efficient infection of the vertebrate host by mosquito bite.

### The interaction with mammalian G3BP and mosquito Rasputin depends on the presence of FGDF motifs in the HVD of chikungunya virus nsP3

**Fig 6:**
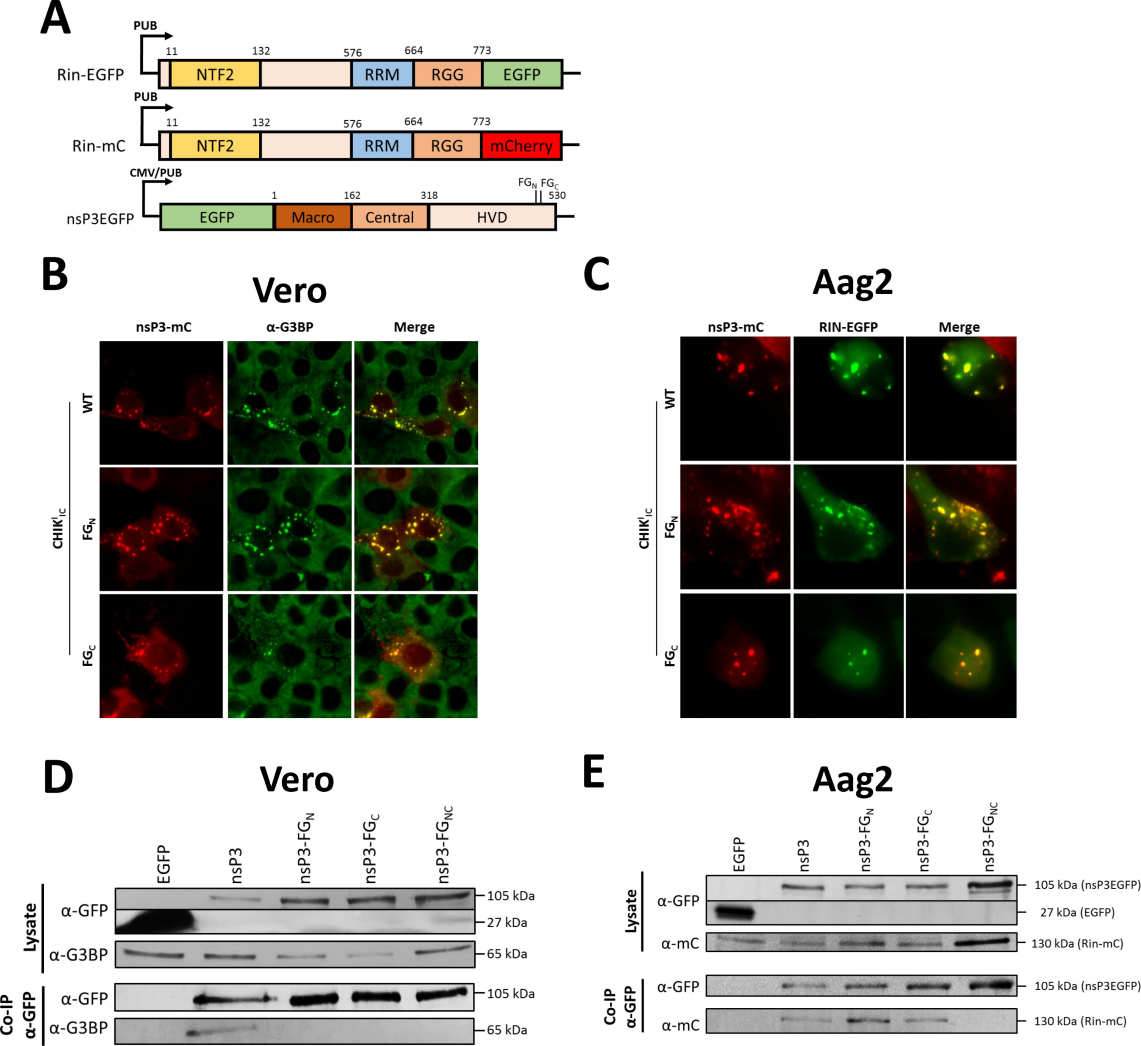
At least one FGDF motif is required for the interaction of nsP3 with G3BP and mosquito Rin. (A) Schematic overview of the used plasmids expressing Rasputin (Rin) fused to EGFP Rin-EGFP or mCherry Rin-mC and CHIKV nsP3 EGFP fusion proteins of wild type and FGDF single- and double-mutant nsP3. CMV cytomegalovirus promoter; PUB *Aedes aegypti* poly-ubiquitin promoter; NTF2 nuclear transport factor 2-like domain; RRM RNA recognition motif; RGG arginine glycine rich region; HVD hypervariable domain. (B) Vero cells were infected with CHIK_IC_nsP3mC, CHIK_IC_nsP3mC-FG_N_ or CHIK_IC_nsP3mC-FG_C_. At 24 hours post infection (hpi) cells were fixed, permeabilized, stained with α-G3BP and visualized by fluorescent microscopy. (C) Aag2 cells were transfected with pPUB-Rin-EGFP and at 24 hours post transfection (hpt) cells were infected with CHIK_IC_nsP3mC, CHIK_IC_nsP3mC-FG_N_ or CHIK_IC_nsP3mC-FG_C_. At 24 hpi cells were fixed and visualized by fluorescence microscopy. (D) Vero cells were transfected with CMV driven plasmids expressing EGFP, nsP3EGFP, nsP3EGFP-FG_C_, nsP3EGFP-FG_N_ or nsP3EGFP-FG_NC_. At 24 hpt cells were lysed and lysates were subjected to co-immunoprecipitation with α-GFP beads. Lysates and co-precipitates were subjected to western blot with α-G3BP and α-GFP antibodies. (E) Aag2 cells were transfected with PUB driven plasmids expressing EGFP, nsP3EGFP, nsP3EGFP-FG_N_, nsP3EGFP-FG_C_ or nsP3EGFP-FG_NC_ and co-transfected with pPUB-Rin-mC. At 24 hpt cells were lysed and lysates were subjected to co-immunoprecipitation with α-GFP beads. Lysates and co-precipitates were subjected to western blot with α-GFP and α-mCherry antibodies.

We have previously shown that mutations in a single FGDF motif do not abrogate the co-localization of CHIKV nsP3 and the mosquito homolog of G3BP, Rin, in insect cells [34]. However, in mammalian cells it has been suggested that mutation of just the N-terminal FGDF motif of nsP3 already dismantles the interaction with G3BP [17,20]. We therefore investigated the effect of mutating either of the two FGDF motifs on the co-localization of CHIKV nsP3 with G3BP in mammalian cells and Rin in mosquito cells. Because there is no anti-Rin antibody available, Aag2 cells were first transfected with a plasmid expressing an EGFP-tagged Rin (pPUB-Rin-EGFP, Fig 6A). Vero (Fig 6B) and Aag2 (Fig 6C) cells were infected with CHIK_IC_nsP3mC, CHIK_IC_nsP3mC-FG_N_ or CHIK_IC_nsP3mC-FG_C_. At 24 hpi, cells were fixed and permeabilized and Vero cells were immuno-stained for G3BP-1. In both mammalian and mosquito cells mutation of either the N-terminal or the C-terminal FGDF motif did not abrogate the co-localization of nsP3 with G3BP and Rin.

As co-localization observed by fluorescent microscopy does not prove that there is a physical protein-protein interaction we investigated the importance of the FGDF motifs for interaction of nsP3 with G3BP in mammalian cells and Rin in mosquito cells by co-immunoprecipitation (co-IP). First, Vero cells were transfected with CMV-driven plasmids expressing the nsP3EGFP fusion proteins nsP3, nsP3-FG_N_, nsP3-FG_C_, nsP3-FG_NC_ or just EGFP as a control (Fig 6A). At 24 hpt cells were lysed and lysates were subjected to co-IP with α-GFP beads followed by western blot detection of nsP3EGFP and G3BP in both the cell lysates and co-IP samples (Fig 6D). The expression of the nsP3 fusion proteins was similar for nsP3-FG_N_, nsP3-FG_C_ and nsP3-FG_NC_ while expression of wild type nsP3 was lower. Yet after co-IP, G3BP was only enriched for wild type nsP3, despite the presence of less nsP3 in the co-IP fraction. G3BP was not enriched for nsP3-FG_N_, nsP3-FG_C_ or nsP3-FG_NC_, suggesting that nsP3 requires duplicate FGDF motifs to establish high-affinity for G3BP in mammalian cells.

Next, Aag2 cells were transfected with PUB-driven plasmids expressing the nsP3EGFP fusion proteins nsP3, nsP3-FG_C_, nsP3-FG_N_ or nsP3-FG_NC_ and co-transfected with pPUB-Rin-mC, encoding a Rin-mCherry fusion protein (Fig 6A). Cells were lysed, subjected to co-IP with α-GFP beads followed by detection of nsP3EGFP and Rin-mC in the cell lysates and precipitated samples (Fig 6E). Expression of the nsP3EGFP fusion proteins was similar for nsP3, nsP3-FG_N_, nsP3-FG_C_, nsP3-FG_NC._ Expression of Rin-mC was similar for nsP3, nsP3-FG_N_, nsP3-FG_C_ transfected samples, while Rin-mC expression was highest in cells transfected with nsP3-FG_NC_. Similar amounts of nsP3, nsP3-FG_N_ and nsP3-FG_C_ but more nsP3-FG_NC_ was precipitated. Interestingly, however, Rin-mC was only enriched for nsP3, nsP3-FG_N_ and nsP3-FG_C_, but not for nsP3-FG_NC_. This demonstrates that nsP3 requires only one FGDF motif for interaction with Rin. These results suggest that differences in the affinities of FGDF motifs for G3BP and Rin may underlie the difference for the required number of FGDF motifs between mammalian and mosquito cells.

## Discussion

The delicacy of alphavirus-vector interactions has been well-illustrated by the discovery that a single A→V mutation in the CHIKV E1 envelope protein could change the viral vector specificity from *Ae*. *aegypti* to *Ae*. *albopictus* [5]. Additionally, for VEEV a single amino acid substitution in the E2 protein could determine its potential to infect *Ochlerotatus taeniorhynchus* mosquitoes [41]. Here, we investigated the importance of the conserved P-rich and FGDF motifs in the HVD of CHIKV nsP3 for infection of mosquito cells and transmission by *Ae*. *aegypti* mosquitoes.

Deletion of the P-rich motif in the nsP3 HVD renders CHIKV unable to replicate in both mammalian and mosquito cells, while amino acid substitutions of the core residues in the P-rich motif resulted in a significant decrease of replication in both cell types. This is similar to previous findings with SINV where deletions in the SINV nsP3 HVD that disrupt the P-rich motif were reported to decrease growth kinetics and plaque size in C7/10 mosquito cells [31], an indication for reduced replication potency or cytopathicity, or both. However, mutations of the P-rich motif did not affect CHIKV infection or transmission by *Ae*. *aegypti* mosquitoes. The nsP3 P-rich motif has high affinity for SH3-domain containing proteins and is known to interact with amphiphysin-I/II [18,23] in mammalian cells, and proposedly also with the *Drosophila* homolog of amphiphysin [18], although no evidence has so far been reported for this interaction in insect cells. We indeed demonstrate that mutation of the P-rich motif results in decreased virus production in mosquito cells, which suggests that nsP3 interacts with mosquito SH3-domain containing proteins, although these interactions do not appear to be essential for transmission by the mosquito vector. It was previously shown that deletions of the P-rich motif of SFV and SINV result in decreased affinity for amphiphysins and lowered replication efficiencies in mammalian cells [18]. Therefore, the decrease in replication of CHIK_IC_^P398A^ and CHIK_IC_^PPR401AAA^ in mammalian cells may be due to reduced affinity for amphiphysins or other cellular proteins that contain an SH3 domain. Of note, both the P398A and PPR401AAA mutations do not mutate all prolines in the CHIKV PxxPPR motif, thus the remaining prolines may still partially interact with host proteins. This may underlie the strong attenuation of CHIK_IC_^P398A^ and CHIK_IC_^PPR401AAA^ after low MOI infection, but not after high MOI infection where the remaining proline(s) may be sufficient to establish successful replication. Complete inactivation of CHIKV after deletion of the entire P-rich motif implies that such interactions are crucial to establish successful virus infection. It has been demonstrated that the arginine residues downstream of the P-rich motif of CHIKV, which are not conserved in SFV and SINV nsP3, partially determine the affinity for amphiphsyin-2 [23]. This might explain why deletion of the whole P-rich motif, including the C-terminal arginines downstream of PxxPPR completely abrogates CHIKV replication, while mutating just the core residues in the P-rich motif results in a less dramatic effect.

Mutation of both FGDF motifs together fully abrogated CHIKV replication in both mammalian and mosquito cells. This indicates that the FGDF motifs not only play an important role during infection of mammalian cells, as previously reported [17,19–22,30], but that they also determine the successful infection of mosquito cells. Furthermore, we show for CHIKV that a single FGDF motif is sufficient to infect mammalian cells, although the replication and infection efficiencies are dramatically decreased compared to wild type CHIKV. In contrast, in mosquito cells, CHIK_IC_-FG_N_ and CHIK_IC_-FG_C_ replicated similarly to the wild type CHIK_IC_ and were efficiently transmitted by *Ae*. *aegypti* mosquitoes after an infectious bloodmeal. This indicates that CHIKV requires only a single FGDF motif for infection of the mosquito vector. Thus, duplicate FGDF motifs appear to be required for the efficient infection from the mosquito’s saliva to a subsequent vertebrate host, which supports the conservation of duplicate FGDF motifs for most mosquito-borne alphaviruses.

The mosquito protein Rin, a homolog of mammalian G3BP, was previously identified as an important regulator of CHIKV transmission, as knock-down of Rin severely decreased the transmission of CHIKV by *Ae*. *albopictus* mosquitoes [34]. In our study, we confirmed that, during CHIKV infection, a single FGDF motif is sufficient for the co-localization of nsP3 with Rin, similar to previous experiments with transiently expressed nsP3 [34]. Mutation of both FGDF motifs in nsP3 together abolished its ability to co-localize and interact with Rin, suggesting that the inability of CHIKV_IC_-FG_NC_ to replicate in mosquito cells is due to absence of the nsP3-Rin interaction. Furthermore, we demonstrate that only one FGDF motif is required for the interaction between nsP3 and Rin, in contrast to mammalian cells where the interaction between nsP3 and G3BP was only observed for wild type nsP3 and not for nsP3 with one mutated FGDF motif. For SFV nsP3 it has been shown that mutation of just the phenylalanine of FG_N_ largely abrogates the interaction with G3BP while mutating the phenylalanine of FG_C_ only decreases the affinity of nsP3 for G3BP [20]. We mutated not only the first phenylalanine of FG_C_ but also the glycine to alanine, both of which have been shown to be important for G3BP binding [40]. This may explain why we could not observe an interaction between G3BP and either nsP3-FG_N_ or nsP3-FG_C_.

G3BP can be present in monomeric, dimeric and polymeric forms [42] and the two FGDF motifs of alphavirus nsP3 each bind one NTF2-like domain of two separate G3BP monomers [20,40]. As a result, mutation of one FGDF motif results in decreased affinity for G3BP and attenuates alphavirus replication [17,20,21]. It is uncertain whether mosquito Rin forms a dimer, although the crystal structure of the *Drosophila* Rin NTF2-like domain was demonstrated in dimeric form [43]. Thus, a single FGDF motif might be sufficient for the interaction with monomeric Rin to support CHIKV replication in mosquito cells and in *Ae*. *aegypti* mosquitoes. Alternatively, the FGDF motifs of nsP3 might have a higher affinity for Rin as compared to G3BP, resulting in sufficient recruitment of Rin to replication complexes in mosquito cells with only a single FGDF motif. As Vero and Aag2 cells were cultured at 37°C and 28°C respectively, lowered affinity of the FGDF motifs for G3BP as compared to Rin may partially be explained by the higher incubation temperature. Potentially, the higher temperature at which mammalian Vero cells are cultured decreases the stability of the nsP3-G3BP interaction, thus resulting in lower affinity of nsP3 for G3BP as compared to Rin.

The exact reason for recruitment of Rin/G3BP by nsP3 is still speculative, although several hypotheses such as recruitment of viral gRNA followed by shielding of the viral replication complex from degrading enzymes [17] and prevention of antiviral stress granule formation [13,21] have been brought forward. Possibly, the interaction with G3BP/Rin is particularly crucial early after infection and a certain level of G3BP/Rin has to be bound by nsP3 to successfully form viral replication complexes. Interestingly, while arthritogenic-alphaviruses recruit G3BP via their FGDF motifs, neurotropic alphaviruses lack FGDF motifs and instead recruit members of the Fragile X syndrome (FXR) family via a different motif in the nsp3 HVD [17,19]. Proteins of the FXR family are abundantly expressed in neuronal cells [44], which may therefore be more susceptible to infection by alphaviruses that use FXR family proteins for their replication. Whether a direct correlation exists between the use of FXR or G3BP for replication and the symptoms caused by alphavirus infection should be the focus of future research. Mosquitoes possess a homolog of one FXR family protein, called Fragile X syndrome-related protein 1 (FMR1). Based on our results, we hypothesize that the nsP3 of neurotropic alphaviruses may interact with mosquito FMR1, similarly to the CHIKV nsP3-Rin interaction. Future research should investigate the possibility of an interaction between nsP3 and mosquito FMR1 and its importance for the transmission of neurotropic alphaviruses by mosquitoes.

Concluding, our results increase the understanding of the functionality of conserved motifs in the alphavirus nsP3 HVD in the context of the arboviral dual-host life cycle. Understanding the underlying mechanisms of G3BP/Rin recruitment by alphavirus nsP3 remains the focus of future research and may well contribute to the development of antiviral drugs and compounds that target this crucial interaction.

## Supporting information

S1 Fig Verification of CHIK_IC_ nsP3 mutants *in vivo* in *Ae*. *aegypti* mosquitoes. RNA was isolated from the bodies of mosquitoes with CHIKV-positive saliva. The nsP3 gene was amplified by RT-PCR and digested with SacII, NotI or ApeKI to screen for preservation of the expected mutations.
(PDF)

## References

[1] De Figueiredo MLG, Figueiredo LTM. Emerging alphaviruses in the americas: Chikungunya and mayaro. Rev Soc Bras Med Trop. 2014;47: 677–683. 10.1590/0037-8682-0246-2014 25626645 10.1590/0037-8682-0246-2014

[2] Esposito DLA, Fonseca BAL da. Will Mayaro virus be responsible for the next outbreak of an arthropod-borne virus in Brazil? Brazilian J Infect Dis. 2017;21: 540–544. 10.1016/j.bjid.2017.06.002 28688628 10.1016/j.bjid.2017.06.002 PMC9425496

[3] Burt FJ, Chen W, Miner JJ, Lenschow DJ, Merits A, Schnettler E, et al Chikungunya virus: an update on the biology and pathogenesis of this emerging pathogen. Lancet Infect Dis. 2017;17: e107–e117. 10.1016/S1473-3099(16)30385-1 28159534 10.1016/S1473-3099(16)30385-1

[4] PAHO. Number of reported cases of chikungunya fever in the Americas 2016 PAHO/WHO 2017.

[5] Tsetsarkin K a, Vanlandingham DL, McGee CE, Higgs S. A single mutation in chikungunya virus affects vector specificity and epidemic potential. PLoS Pathog. 2007;3: e201 10.1371/journal.ppat.0030201 18069894 10.1371/journal.ppat.0030201 PMC2134949

[6] Strauss JH, Strauss EG. The alphaviruses: gene expression, replication, and evolution. Microbiol Rev. 1994;58: 491–562. 7968923 10.1128/mr.58.3.491-562.1994 PMC372977

[7] Rupp JC, Sokoloski KJ, Gebhart NN, Hardy RW. Alphavirus RNA synthesis and non-structural protein functions. J Gen Virol. 2015;96: 2483–2500. 10.1099/jgv.0.000249 26219641 10.1099/jgv.0.000249 PMC4635493

[8] Pietilä MK, Hellström K, Ahola T. Alphavirus polymerase and RNA replication. Virus Res. 2017; 1–14. 10.1016/j.virusres.2017.01.007 28104453 10.1016/j.virusres.2017.01.007

[9] Götte B, Liu L, McInerney G. The enigmatic alphavirus non-structural protein 3 (nsP3) revealing its secrets at last. Viruses. 2018;10: 105 10.3390/v10030105 29495654 10.3390/v10030105 PMC5869498

[10] Fros J, Pijlman G. Alphavirus infection: host cell shut-off and inhibition of antiviral responses. Viruses. 2016;8: 166 10.3390/v8060166 27294951 10.3390/v8060166 PMC4926186

[11] Remenyi R, Roberts GC, Zothner C, Merits A, Harris M. SNAP-tagged chikungunya virus replicons improve visualisation of non-structural protein 3 by fluorescence microscopy. Sci Rep. 2017;7: 5682 10.1038/s41598-017-05820-0 28720784 10.1038/s41598-017-05820-0 PMC5515888

[12] Ehsani N, Vihinen H, Kujala P, Ika A. Biogenesis of the Semliki Forest virus RNA replication complex. J Virol. 2001;75: 3873–3884. 10.1128/JVI.75.8.3873-3884.2001 11264376 10.1128/JVI.75.8.3873-3884.2001 PMC114878

[13] Fros JJ, Domeradzka NE, Baggen J, Geertsema C, Flipse J, Vlak JM, et al Chikungunya virus nsP3 blocks stress granule assembly by recruitment of G3BP into cytoplasmic foci. J Virol. 2012;86: 10873–10879. 10.1128/JVI.01506-12 22837213 10.1128/JVI.01506-12 PMC3457282

[14] Gorchakov R, Garmashova N, Frolova E, Frolov I. Different types of nsP3-containing protein complexes in Sindbis virus-infected cells. J Virol. 2008;82: 10088–101. 10.1128/JVI.01011-08 18684830 10.1128/JVI.01011-08 PMC2566286

[15] Peranen J, Takkinen K, Kalkkinen N, Kaariainen L. Semliki Forest virus-specific non-structural protein nsP3 is a phosphoprotein. J Gen Virol. 1988;69: 2165–2178. 10.1099/0022-1317-69-9-2165 2970523 10.1099/0022-1317-69-9-2165

[16] Liang Z, Li G. Recombinant Sindbis virus expressing functional GFP in the nonstructural protein nsP3. Gene Ther Mol Biol. 2005;9: 317–324.

[17] Kim DY, Reynaud JM, Rasalouskaya A, Akhrymuk I, Mobley JA, Frolov I, et al New world and old world alphaviruses have evolved to exploit different components of stress granules, FXR and G3BP proteins, for assembly of viral replication complexes. PLoS Pathog. 2016;12: 1–31. 10.1371/journal.ppat.1005810 27509095 10.1371/journal.ppat.1005810 PMC4980055

[18] Neuvonen M, Kazlauskas A, Martikainen M, Hinkkanen A, Ahola T, Saksela K. SH3 domain-mediated recruitment of host cell amphiphysins by alphavirus nsp3 promotes viral RNA replication. PLoS Pathog. 2011;7 10.1371/journal.ppat.1002383 22114558 10.1371/journal.ppat.1002383 PMC3219718

[19] Frolov I, Kim DY, Akhrymuk M, Mobley JA, Frolova EI. Hypervariable domain of Eastern equine encephalitis virus nsP3 redundantly utilizes multiple cellular proteins for replication complex assembly. J Virol. 2017;91: e00371–17. 10.1128/JVI.00371-17 28468889 10.1128/JVI.00371-17 PMC5487569

[20] Schulte T, Liu L, Panas MD, Thaa B, Dickson N, Achour A, et al Combined structural, biochemical and cellular evidence demonstrates that both FGDF motifs in alphavirus nsP3 are required for efficient replication. Open Biol. 2016;6: 160078 10.1098/rsob.160078 27383630 10.1098/rsob.160078 PMC4967826

[21] Panas MD, Varjak M, Lulla A, Er Eng K, Merits A, Karlsson Hedestam GB, et al Sequestration of G3BP coupled with efficient translation inhibits stress granules in Semliki Forest virus infection. Mol Biol Cell. 2012;23: 4701–4712. 10.1091/mbc.E12-08-0619 23087212 10.1091/mbc.E12-08-0619 PMC3521679

[22] Panas MD, Ahola T, McInerney GM. The C-terminal repeat domains of nsP3 from the old world alphaviruses bind directly to G3BP. J Virol. 2014;88: 5888–5893. 10.1128/JVI.00439-14 24623412 10.1128/JVI.00439-14 PMC4019107

[23] Tossavainen H, Aitio O, Hellman M, Saksela K, Permi P. Structural basis of the high affinity interaction between the Alphavirus nonstructural protein-3 (nsP3) and the SH3 domain of amphiphysin-2. J Biol Chem. 2016;291: 16307–16317. 10.1074/jbc.M116.732412 27268056 10.1074/jbc.M116.732412 PMC4965578

[24] Malet H, Coutard B, Jamal S, Dutartre H, Papageorgiou N, Neuvonen M, et al The crystal structures of chikungunya and Venezuelan equine encephalitis virus nsP3 macro domains define a conserved adenosine binding pocket. J Virol. 2009;83: 6534–6545. 10.1128/JVI.00189-09 19386706 10.1128/JVI.00189-09 PMC2698539

[25] Shin G, Yost SA, Miller MT, Elrod EJ, Grakoui A, Marcotrigiano J. Structural and functional insights into alphavirus polyprotein processing and pathogenesis. Proc Natl Acad Sci. 2012;109: 16534–16539. 10.1073/pnas.1210418109 23010928 10.1073/pnas.1210418109 PMC3478664

[26] Vihinen H. Phosphorylation site analysis of Semliki Forest virus nonstructural protein 3. J Biol Chem. 2000;275: 27775–27783. 10.1074/jbc.M002195200 10851234 10.1074/jbc.M002195200

[27] Li G, La Starza MW, Reef Hardy W, Strauss JH, Rice CM. Phosphorylation of Sindbis virus nsP3 in vivo and in vitro. Virology. 1990;179: 416–427. 10.1016/0042-6822(90)90310-N 2145690 10.1016/0042-6822(90)90310-n

[28] Strauss EG, Levinson R, Rice CM, Dalrymple J, Strauss JH. Nonstructural proteins nsP3 and nsP4 of Ross River and O’Nyong-nyong viruses: Sequence and comparison with those of other alphaviruses. Virology. 1988;164: 265–274. 10.1016/0042-6822(88)90644-7 2834873 10.1016/0042-6822(88)90644-7

[29] Kristensen O. Crystal structure of the G3BP2 NTF2-like domain in complex with a canonical FGDF motif peptide. Biochem Biophys Res Commun. 2015;467: 53–57. 10.1016/j.bbrc.2015.09.123 26410532 10.1016/j.bbrc.2015.09.123

[30] Scholte FEM, Tas A, Albulescu IC, Žusinaite E, Merits A, Snijder EJ, et al Stress granule components G3BP1 and G3BP2 play a proviral role early in chikungunya virus replication. J Virol. 2015;89: 4457–69. 10.1128/JVI.03612-14 25653451 10.1128/JVI.03612-14 PMC4442398

[31] Lastarza-MW, Grakoui-A, Rice-CM. Deletion and duplication mutations in the C-terminal nonconserved region of Sindbis virus nsP3: effects on phosphorylation and on virus replication in vertebrate and invertebrate cells. Virology. 1994;202: 224–232. 10.1006/viro.1994.1338 7912020 10.1006/viro.1994.1338

[32] Lim E, Lee W, Madzokere E, Herrero L. Mosquitoes as suitable vectors for alphaviruses. Viruses. 2018;10: 84 10.3390/v10020084 29443908 10.3390/v10020084 PMC5850391

[33] Saxton-Shaw KD, Ledermann JP, Borland EM, Stovall JL, Mossel EC, Singh AJ, et al O’nyong nyong virus molecular determinants of unique vector specificity reside in non-structural protein 3. PLoS Negl Trop Dis. 2013;7: e1931 10.1371/journal.pntd.0001931 23359824 10.1371/journal.pntd.0001931 PMC3554527

[34] Fros JJ, Geertsema C, Zouache K, Baggen J, Domeradzka N, van Leeuwen DM, et al Mosquito Rasputin interacts with chikungunya virus nsP3 and determines the infection rate in Aedes albopictus. Parasit Vectors. 2015;8: 464 10.1186/s13071-015-1070-4 26384002 10.1186/s13071-015-1070-4 PMC4573678

[35] Göertz G.P.; Vogels C.B.F.; Geertsema C.; Koenraadt C.J.M.; Pijlman G. Mosquito co-infection with Zika and chikungunya virus allows simultaneous transmission without affecting vector competence of Aedes aegypti. PLoS Negl Trop Dis. 2017; 1–22. 10.1371/journal.pntd.0005654 28570693 10.1371/journal.pntd.0005654 PMC5469501

[36] Anderson MAE, Gross TL, Myles KM, Adelman ZN. Validation of novel promoter sequences derived from two endogenous ubiquitin genes in transgenic Aedes aegypti. Insect Mol Biol. 2010;19: 441–449. 10.1111/j.1365-2583.2010.01005.x 20456509 10.1111/j.1365-2583.2010.01005.x PMC3605713

[37] Metz SW, Geertsema C, Martina BE, Andrade P, Heldens JG, van Oers MM, et al Functional processing and secretion of chikungunya virus E1 and E2 glycoproteins in insect cells. Virol J. 2011;8: 1–12. 10.1186/1743-422X-8-1 21762510 10.1186/1743-422X-8-353 PMC3162542

[38] Nasar F, Palacios G, Gorchakov R V., Guzman H, Da Rosa APT, Savji N, et al Eilat virus, a unique alphavirus with host range restricted to insects by RNA replication. Proc Natl Acad Sci. 2012;109: 14622–14627. 10.1073/pnas.1204787109 22908261 10.1073/pnas.1204787109 PMC3437828

[39] Hermanns K, Zirkel F, Kopp A, Marklewitz M, Rwego IB, Estrada A, et al Discovery of a novel alphavirus related to Eilat virus. J Gen Virol. 2017;98: 43–49. 10.1099/jgv.0.000694 28206905 10.1099/jgv.0.000694

[40] Panas MD, Schulte T, Thaa B, Sandalova T, Kedersha N, Achour A, et al Viral and cellular proteins containing FGDF motifs bind G3BP to block stress granule formation. PLoS Pathog. 2015;11: 1–22. 10.1371/journal.ppat.1004659 25658430 10.1371/journal.ppat.1004659 PMC4450067

[41] Brault AC, Powers AM, Ortiz D, Estrada-Franco JG, Navarro-Lopez R, Weaver SC. Venezuelan equine encephalitis emergence: Enhanced vector infection from a single amino acid substitution in the envelope glycoprotein. Proc Natl Acad Sci. 2004;101: 11344–11349. 10.1073/pnas.0402905101 15277679 10.1073/pnas.0402905101 PMC509205

[42] Tourrière H, Chebli K, Zekri L, Courselaud B, Blanchard JM, Bertrand E, et al The RasGAP-associated endoribonuclease G3BP assembles stress granules. J Cell Biol. 2003;160: 823–831. 10.1083/jcb.200212128 12642610 10.1083/jcb.200212128 PMC2173781

[43] Vognsen T, Kristensen O. Crystal structure of the Rasputin NTF2-like domain from Drosophila melanogaster. Biochem Biophys Res Commun. 2012;420: 188–192. 10.1016/j.bbrc.2012.02.140 22414690 10.1016/j.bbrc.2012.02.140

[44] Antar LN, Li C, Zhang H, Carroll RC, Bassell GJ. Local functions for FMRP in axon growth cone motility and activity-dependent regulation of filopodia and spine synapses. Mol Cell Neurosci. 2006;32: 37–48. 10.1016/j.mcn.2006.02.001 16631377 10.1016/j.mcn.2006.02.001

