# Supplementary material for: Conserved motifs in the hypervariable domain of chikungunya virus nsP3 required for transmission by Aedes aegypti mosquitoes
Source: PLoS Negl Trop Dis. 2018 Nov 9;12(11):e0006958. doi: 10.1371/journal.pntd.0006958 (PMC6249005; doi:10.1371/journal.pntd.0006958)
Supplement: S1 Fig — RNA was isolated from the bodies of mosquitoes with CHIKV-positive saliva. The nsP3 gene was amplified by RT-PCR and digested with SacII, NotI or ApeKI to screen for preservation of the expected mutations. (PDF) [file pntd.0006958.s001.pdf]

S1 Fig

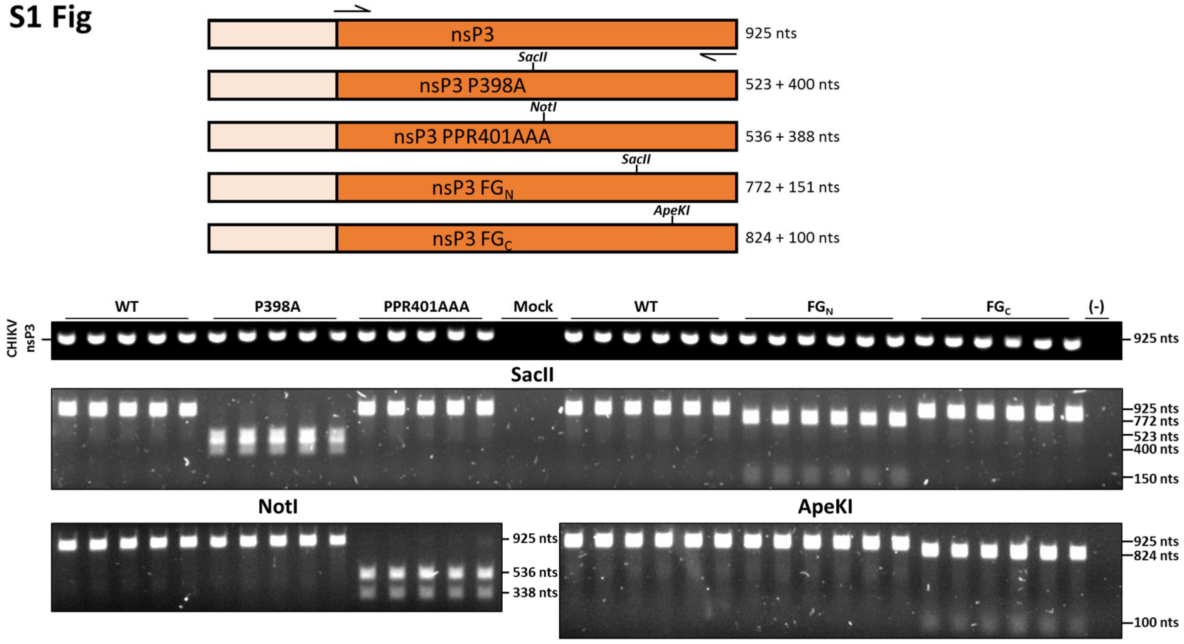

S1. Fig. Verification of CHIK$_{IC}$ nsP3 mutants *in vivo* in *Ae. aegypti* mosquitoes. RNA was isolated from the bodies of mosquitoes with CHIKV-positive saliva. The nsP3 gene was amplified by RT-PCR and digested with SacII, NotI or ApeKI to screen for preservation of the expected mutations.
